# Genetic Variation in the Complete MgPa Operon and Its Repetitive Chromosomal Elements in Clinical Strains of *Mycoplasma genitalium*

**DOI:** 10.1371/journal.pone.0015660

**Published:** 2010-12-20

**Authors:** Liang Ma, Jørgen S. Jensen, Miriam Mancuso, Ryoichi Hamasuna, Qiuyao Jia, Chris L. McGowin, David H. Martin

**Affiliations:** 1 Section of Infectious Diseases, Department of Medicine, Louisiana State University Health Sciences Center, New Orleans, Louisiana, United States of America; 2 Mycoplasma Laboratory, Statens Serum Institut, Copenhagen, Denmark; 3 Department of Urology, University of Occupational and Environmental Health, Yahatanishi-ku, Kitakyushu, Japan; Royal Tropical Institute, The Netherlands

## Abstract

*Mycoplasma genitalium* has been increasingly recognized as an important microbe not only because of its significant association with human genital tract diseases but also because of its utility as a model for studying the minimum set of genes necessary to sustain life. Despite its small genome, 4.7% of the total genome sequence is devoted to making the MgPa adhesin operon and its nine chromosomal repetitive elements (termed MgPars). The MgPa operon, along with 9 MgPars, is believed to play an important role in pathogenesis of *M. genitalium* infection and has also served as the main target for development of diagnostic tools. However, genetic variation in the complete MgPa operon and MgPars among clinical strains of *M. genitalium* has not been addressed. In this study we examined the genetic variation in the complete MgPa operon (approximately 8.5 kb) and full or partial MgPar sequences (0.4–2.6 kb) in 15 geographically diverse strains of *M. genitalium*. Extensive variation was present in four repeat regions of the MgPa operon (with homology to MgPars) among and within strains while the non-repeat regions (without homology to MgPars) showed low-level variation among strains and no variation within strains. MgPars showed significant variation among strains but were highly homogeneous within strains, supporting gene conversion as the likely recombination mechanism. When applying our sequence data to evaluate published MgPa operon-based diagnostic PCR assays and genotyping systems, we found that 11 of 19 primers contain up to 19 variable nucleotides and that the target for one of two typing systems is located in a hypervariable repeat region, suggesting the likelihood of false results with some of these assays. This study not only provides new insights into the role of the MgPa operon in the pathogenesis of *M. genitalium* infection but has important implications for the development of diagnostic tools.

## Introduction

*Mycoplasma genitalium* has been increasingly recognized as an important microbe not only because it causes significant human genital tract disease [1] but also because it has the smallest genome of any known free living microorganism and hence is a model for the study of the minimum set of genes necessary to sustain life [2], [3]. Despite its small genome, 4.7% of the total genomic sequence is devoted to making the MgPa adhesin operon and its repetitive chromosomal sequences, known as MgPars [4]. The MgPa operon has been the most extensively studied molecule of *M. genitalium*. The two adhesin proteins encoded by the MgPa operon are the major surface proteins in this organism and are required in the development of the terminal-organelle structure and attachment of the organism to host epithelial cells [5], [6]. Both proteins are capable of eliciting very strong antibody responses in *M. genitalium* infected patients and experimentally infected animals [5], [7]–[9], suggesting an important role in pathogenesis.

**Figure 1:**
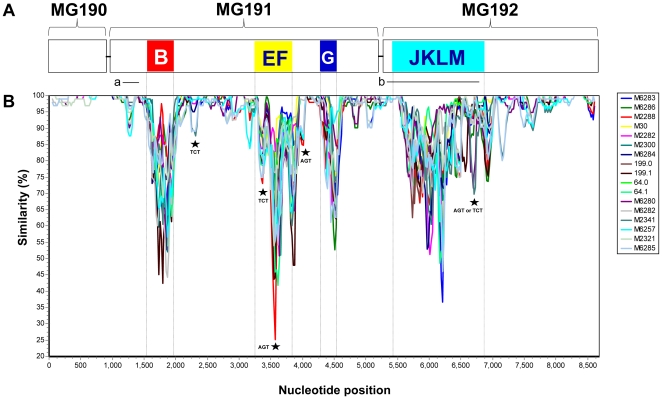
Sequence variability in the full-length MgPa operon of *M. genitalium*. (**A**) Schematic representation of the MgPa operon structure based on the G37 genome sequence (GenBank accession number NC_000908). Regions B, EF, G and JKLM highlighted in colors are referred to in the text as repeat regions, which are homologous to various parts of the nine MgPar regions scattered around the genome as shown in Fig. 2. The two horizontal bars labeled a and b represent the regions used as markers for the genotyping systems described by Hjorth *et al*. [14] and Musatovova *et al*. [26], respectively. (**B**) Similarity plot of the MgPa operon sequences from 15 *M. genitalium* strains as shown in the right. Each curve is a comparison between the strain being analyzed and G37. Each point plotted is the percent identity within a sliding window of 100 bp centered on the position plotted, with a step size between points of 20 bp. Dotted vertical lines indicate borders for the repeat regions. Stars indicate the location of variable trinucleotide tandem repeats, with the repeat units alongside the star. Details on these trinucleotide tandem repeats are to be reported elsewhere (Ma *et al.*, unpublished data).

**Figure 2:**
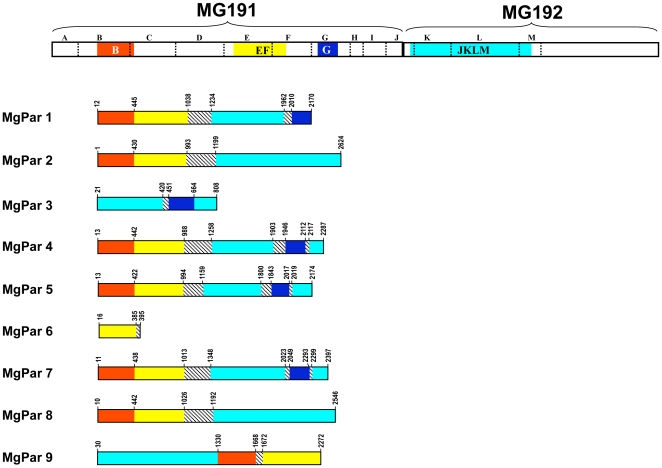
Schematic representation of sequence homology of the MG191 and MG192 genes with MgPars based on the *M. genitalium* G37 genome (GenBank accession number NC_000908). Divisions A through M indicated by dotted vertical lines represent the restriction fragments described previously [13], [31]. The four repeat regions B, EF, G and JKLM are highlighted in colors (consistent with colors used in Fig. 1). Homologous regions between MG191/MG192 and MgPars are indicated in identical colors. The hatched box represents intervening sequences that are unusually A-T rich and contain stop codons. The numbers bordering each segment of each of the MgPars refer to the nucleotide positions in the full-length MgPars as described elsewhere [10]. The line length in the diagrams is not always proportional to the number of nucleotides involved due to the presence of minor deletions/insertions. MgPars found in clinical strains involved in this study showed the same architecture but divergent sequences (with different coordinates for each region shown) compared to G37 MgPars.

Based on studies of the G37 type strain [4], the MgPa operon consists of three genes in the order of MG190 (*mgpA*), MG191 (*mgpB*) and MG192 (*mgpC*) (Figure 1A). The expression site for each of these three genes is present in only one copy per genome while there are nine distinct MgPar sequences dispersed throughout the genome (Figure 2). MgPar sequences contain partial copies of the MG191 and MG192 genes. There are three MgPar homologous regions within the MG191 gene, designated herein as repeat regions B, EF and G [1], [10], whereas there is one large MgPar homologous region within the MG192 gene, designated herein as repeat region JKLM [11]. The remaining regions in all three genes of the MgPa operon that have no homology to any MgPar sequences are referred as non-repeat regions. Previous studies have demonstrated that extensive variation occurs in MG191 and MG192 repeat regions within and among *M. genitalium* strains [1], [10]–[13]. Such variation can be explained by homologous recombination of MgPars with the MG191 and MG192 expression sites [11], [12]. However it remains unclear whether or not gene crossover and gene conversion have occurred alone or simultaneously in vitro and in vivo; information is also lacking about the recombination machinery and how it is regulated in this organism with a markedly reduced genome. Regardless of the recombination mechanisms, it has been generally accepted that genetic variation in MG191 and MG192 repeat regions represent a mechanism used by *M. genitalium* to evade the host immune response and to adapt to diverse host microenvironments, thus establishing persistent infection.

In contrast to studies of variation in the MgPa repeat regions, there has been less effort to study variation in MgPa non-repeat regions and MgPar loci. The sequences contained in the MgPa non-repeat regions account for 68% of the total MgPa operon sequence. Only a few very short non-repeat regions in the MG191 and MG192 genes have been investigated for their variability in clinical strains [14]–[16] and as yet no information is available about the mechanism or function of this variation. Nevertheless the MG191 gene has served as the main target for development of diagnostic PCR assays [15], [17]–[20] and genotyping methods [14]. As for the MgPars, besides the G37 type strain [4], there is no complete set of MgPar sequences available from a clinical strain. It remains unknown how MgPars differ from each other among clinical strains and whether they share the same architecture as the G37 MgPars. Detailed studies of these questions would provide insights into MgPa recombination and variation and contribute to a better understanding of how *M. genitalium* maintains persistent reproductive tract infection.

Study of *M. genitalium* genetic variation has been hampered by the limited availability of genetic materials due to difficulties in isolation and cultivation of this organism from clinical specimens. Although there are several isolates of *M. genitalium* commercially available from the American Type Culture Collection, they are all very closely related to the G37 type strain and possibly could be the same strain based on sequence analysis of multiple genomic loci [11], [14], [21], [22]. In this study we took advantage of the availability of about a dozen *M. genitalium* isolates from our previous studies [23]–[25], which allowed us to do extensive genetic analyses. The goals of this study were to investigate the sequence variability of the whole MgPa operon and MgPars in *M. genitalium* clinical strains, and to explore the implications of variability for their roles in pathogenesis and the development of molecular diagnostic methods.

## Results

### Variation of the whole MgPa operon among clinical strains

The complete MgPa operon was amplified in up to six overlapping fragments from all specimens studied (Table 1). By direct sequencing of the PCR products, homogeneous sequences for each specimen were observed except for the four repeat regions (including B, EF, G and JKLM regions) and two non-repeat regions containing trinucleotide tandem repeats (TTRs, Figure 1). Analysis of 3–18 plasmid clones for each fragment showing mixed sequences in repeat regions identified 2 or 3 variants as follows: region B in strains M2282 and M2321, region EF in 64.0 and 64.1, region G in strains M2321 and 199.1, and region JKLM in strains M2282, M2300, and clinical specimens 199.0 and 199.1. To assemble the MgPa operon using fragments containing multiple variants, only the most predominant sequence was selected.

**Table 1:** Sequence variation of the MgPa operon among *M. genitalium* axenic isolates and urine specimens.

Specimen	Origin (reference)	Size (bp)/nucleotide difference (%) compared to G37^a^			
		MgPa operon	MG190	MG191	MG192
Axenic isolates					
M30	U.K. [32]	8446/3.3	957/0	4332/5.6	3150/4.5
M2282	Denmark [25]	8455/3.2	957/0.1	4332/5.3	3159/4.9
M2288	Denmark [25]	8491/5.1	957/0.5	4380/8.7	3147/8.2
M2300	Denmark [25]	8455/3.1	957/0	4326/5.9	3165/4.3
M2321	Denmark [25]	8470/4.4	957/1.1	4344/8.6	3162/6.0
M2341	Denmark [25]	8467/4.6	957/0	4359/6.5	3144/7.8
M6257	Sweden [33]	8464/4.9	957/0.6	4326/7.8	3174/7.2
M6280	Sweden [33]	8437/4.6	957/0.6	4335/7.1	3138/7.5
M6282	Japan [23]	8470/4.8	957/0.5	4344/8.0	3162/7.4
M6283	Japan [23]	8443/4.7	957/0.6	4344/8.1	3135/7.2
M6284	Japan [23]	8434/3.4	957/0	4329/5.4	3141/4.9
M6285 (or R65G)	Sweden [34]	8458/5.6	957/0.5	4323/9.3	3171/8.9
M6286 (or R67G)	Sweden [34]	8473/5.3	957/1.0	4353/8.4	3156/7.9
Urine specimens^b^					
199.0	U.S.A. [11]	8434/5.3	957/0.5	4323/8.3	3147/7.5
199.1	U.S.A. [11]	8434/5.2	957/0.5	4323/8.6	3147/7.6
64.0	U.S.A. [11], [16]	8506/4.0	957/0.6	4359/7.3	3183/7.0
64.1	U.S.A. [11], [16]	8497/4.5	957/0.6	4350/7.6	3183/7.4

a ) Difference includes nucleotide substitutions, deletions and additions in the coding region compared to the published G37 sequence with 8458 bp in MgPa operon, 957 bp in MG190, 4335 bp in MG191 and 3162 bp in MG192 (GenBank accession number NC_000908).

b ) Specimens 199.0 and 199.1 were from the patient no. 199 at the first visit and a 10-day follow-up visit, respectively, whereas specimens 64.0 and 64.1 were from the patient no. 64 at the first visit and a 11-day follow-up visit, respectively.

The length of the assembled complete MgPa operon varied from 8,434 bp to 8,506 bp (Table 1). The order of the three genes encoded by the MgPa operon in all strains is the same as in the G37 type strain. All operon sequences differed from each other and also from the published G37 operon sequence at both the nucleotide and deduced amino acid levels. The nucleotide difference of the whole operon varied from 4% to 8% in pair-wise comparisons between these strains. Repeat regions B, EF, G and JKLM showed strikingly greater variation than non-repeat regions (Figure 1). Of the four repeat regions the MG191 repeat region B had the highest variation, with a difference of 15–25% (median  = 20%) at the nucleotide level and 26–43% (median  = 37%) at the deduced amino acid level and the MG192 repeat region JKLM had the lowest variation, with a difference of 9–15% (median  = 14%) at the nucleotide level and 18–29% (median  = 24%) at the deduced amino acid level (Table 2). In contrast to repeat regions, non-repeat regions showed much less variation, with a difference varying from 0–1.4% to 0–10.4% at the nucleotide level and 0–0.9% to 0–9.8% at the deduced amino acid level in pair-wise comparison between strains. The complete MG190 gene showed the least variability with single nucleotide polymorphisms in only 14 positions scattered over its full-length, with 11 of them containing synonymous (silent) nucleotide changes. The most variable region was the non-repeat region between the MG191 repeat regions EF and G, which was approximately 400 bp in size and showed single nucleotide polymorphisms in 48 positions and a tandem repeat motif (AGT) varying in the number of repeat from 5 to 12. Clustering tree analysis based on complete MgPa operon sequences or individual repeat or non-repeat regions did not show any clustering associated with geographic origin of the strain (data not shown).

**Table 2:** Sequence variation in the MG191 and MG192 repeat regions.

Specimen	Nucleotide/amino acid difference (%) compared to G37^a^			
	MG191-B(575–1016)	MG191-EF(2292–2876)	MG191-G(3305–3550)	MG192-JKLM(126–1548)
M30	19.7/36.6	15.5/30.5	14.2/22.2	9.7/19.7
M2282	20.1/43.7	11.3/22.2	20.3/37.0	10.2/21.4
M2288	15.7/31.7	21.0/39.5	27.1/35.0	15.8/27.3
M2300	19.9/37.1	16.7/35.6	12.9/25.3	9.1/18.9
M2321	21.9/30.3	17.7/31.8	16.9/31.2	12.0/18.6
M2341	18.3/32.2	17.9/36.8	10.2/25.3	13.0/24.2
M6257	20.9/33.3	17.7/34.4	9.5/13.4	13.6/25.3
M6280	19.3/32.8	18.0/36.9	11.9/22.5	14.3/27.1
M6282	25.2/43.0	17.0/30.9	20.3/31.2	14.7/25.9
M6283	21.5/33.8	16.4/31.1	17.6/30.4	13.5/21.8
M6284	18.1/37.9	15.0/31.0	14.2/30.0	10.4/22.5
M6285	22.4/31.5	21.0/39.1	20.0/35.0	13.8/25.3
M6286	19.9/26.9	15.9/27.4	22.0/30.0	15.1/29.8
199.0	23.0/37.0	19.9/39.7	19.7/32.9	14.6/26.7
199.1	23.0/37.0	19.9/39.7	22.0/33.8	14.7/26.8
64.0	20.1/39.3	14.0/26.0	18.3/30.0	11.1/20.6
64.1	20.1/39.3	18.6/36.3	18.3/30.0	11.9/22.1

a ) Numbers in parentheses below each region refer to the nucleotide positions relative to the presumed MG191 and MG192 translational start site based on the published G37 genome (GenBank accession number NC_000908).

### Architecture and variation of MgPar sequences

We report here the full-length sequence of all nine MgPar loci from patient 199 (GenBank accession numbers EF117293-EF117301). These data represent the only complete set of MgPar sequences available to date from a clinical strain other than the type strain G37. These MgPars showed the same architecture but divergent sequence compared to that reported for the G37 MgPars [1], [10], [11], [13]. The order of the regions homologous to the MG191 and MG192 repeat regions was the same as in the G37 MgPars (Figure 2). All MgPars from the patient specimens showed features similar to the G37 MgPars as previously described [13], including the presence of AT-rich sequences with stop codons (Figure 2), the maintenance of partial ORFs in individual regions with homology to the MG191 and MG192 genes, and the occurrence of insertions and deletions in multiples of three nucleotides. Such sequence characteristics were also noted in complete MgPars 3, 6, 8 and 9 and partial MgPars 1, 2, 4, 5 and 7 sequences in the two sequential specimens from patient 64, and partial MgPars 1, 2, 8 and 9 in five Danish isolates. All these observations are consistent with the notion that MgPar sequences are not directly expressed as proteins unless they are recombined into the expression sites of the MG191 or MG192 gene [1], [10]–[13].

We studied a total of 43 MgPar loci, with 26 of them sequenced from plasmid clones. Aside from the TTR copy number variation, all 43 MgPars showed homogeneous sequences except for the MgPar 7 in specimen 64.0 as described below. All these MgPars differed from each other by 2–25% of nucleotides and also from those of G37 by 12–23% of nucleotides. No MgPars were identical between any two strains.

### Intrastrain variation and recombination of the MgPa operon and MgPars

**Figure 3:**
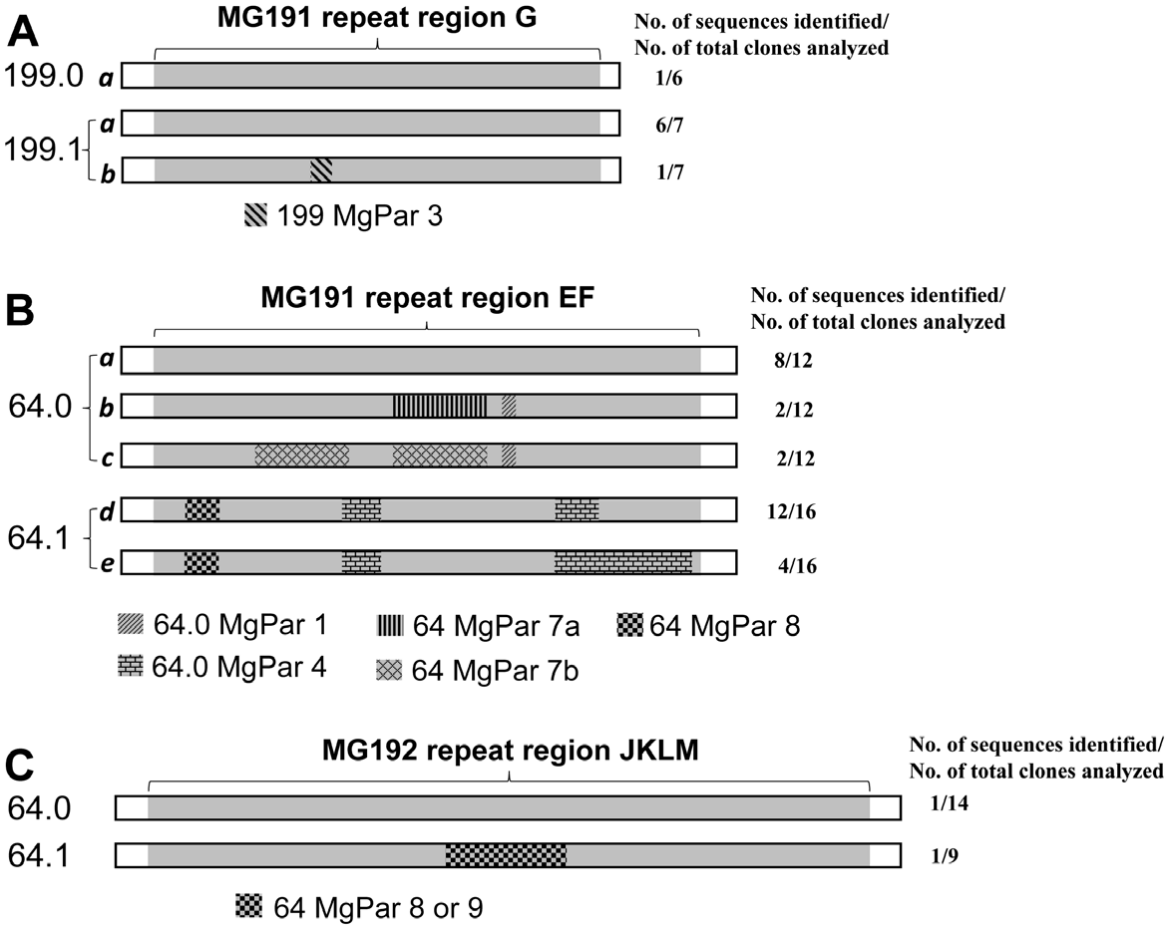
Sequence shifts in MG191 and MG192 repeat regions in two sequential urine specimens from each of two *Mycoplasma genitalium*–infected patients (199 and 64, **Table 1**). (**A**) In patient 199, sequence shifts were identified in MG191 repeat region G as shown here as well as MG192 repeat region JKLM as described elsewhere [11]. The sequence change between these two variant sequences in repeat region G (*a* and *b*) could be explained by homologous recombination with MgPar 3, which was identical between the first- and second-visit specimens. (**B**) Sequence shifts in the MG191 repeat region EF in patient 64. (**C**) Sequence shift in the MG192 repeat region JKLM in patient 64. Except for two single base substitutions in (B) all sequence changes could be explained by homologous recombination with a MgPar sequence found in the first- and/or the second-visit specimen as shown. MgPars 2, 7, 8 and 9 were obtained from both specimens with MgPars 2, 8 and 9 being identical between the first- and second-visit specimens while all remaining MgPars were obtained only from one specimen. Each repeat region along with its upstream and downstream conserved regions was amplified as one fragment and sequenced after subcloning, with the number of plasmid clones analyzed shown on the right of each bar. Sequence changes in each variant and their matching MgPars are indicated by texture. The sequences for the two variants of MG191 repeat region in *A*, and five variants of MG191 repeat region EF (*a* through *e*) and 2 variants of MgPar 7 (7a and 7b) in *B* are available under GenBank accession numbers HQ011255-61, FJ872565 and FJ872566. The remaining accession numbers are listed in Methods.

Analysis of the whole MgPa operon revealed no sequence change in any non-repeat or non-TTR regions between the first- and second-visit specimens with a 10–11 day interval from each of the two New Orleans patients while sequence shifts between the first- and second-visit specimens were observed in two of the four repeat regions in each (Figure 3). Studies of nine complete MgPars in two sequential specimens from patient 199 and six complete or partial MgPars in two sequential specimens from patient 64 showed no sequence changes (excluding the TTR copy number variation) for any MgPar within or between the first- and second-visit specimen except for the MgPar 7 in two specimens 64.0 and 64.1 from patient 64. Sequence changes in all MG191 and MG192 repeat regions from both patients' specimens could be explained by homologous recombination with MgPars (Figure 3).

### Evaluation of the sequences of the MgPa regions used in published diagnostic PCR and genotyping assays

By mapping the 19 previously-reported primers used in diagnostic PCR assays against the sequence alignment of the MgPa operon from the published G37 type strain and the 15 strains sequenced in this study, we found that 17 of them are located in the non-repeat regions and the remaining 2 located in the MG191 repeat region B (Table 3). Nine of the 17 primers located in the non-repeat regions contain nucleotides in 1 to 5 variable positions while the two primers located in the MG191 repeat region B harbor nucleotides in 11 and 19 variable sites, respectively. Of the two published MgPa-based genotyping systems, one [14] used a target at the 5′-end, non-repeat region of the MG191 gene and the other [26] used a target including almost the entire MG192 JKLM repeat region (Figure 1).

**Table 3:** Sequence variation in published PCR primers targeting the MgPa operon.

Study	Forward primer^a^ (5′-3′)	Reverse primer a (5′-3′)
Jensen *et al*., 1991 [18]	MgPa-1, AGTTGATGAAACcTTAACCCCTTGG	MgPa-3, CCGTTGAGGGGTTTTCCATTTTTGC
Palmer *et al*., 1991 [20]	Mg1 (outer), TGTCtATgAcCAGTATGTACMg3 (inner), GTAATTAGTTACTCAGTAGA	Mg2, CtGCTTTGGTCAAgACATCA
Jensen *et al*., 1993 [28]	MgPa-476, ATGGcGAGCCTATCTTTGATCCTTTTAA	MgPa-903, TTCacctccccaCTaCTgtccTtATgc
Cadieux *et al*., 1993 [17]	G3A, GCTTTAAAcCTGGTAACCAGATTGACT	G3B, GAGCGTTAGAGaTCCCTGTTCTGTTA
de Barbeyrac, *et al*., 1993 [27]	MGS-1, GAGCCTTTCTAACCGCTGC	MGS-4, GTTGTTATCATACCTTCTGATMGS-2, GTgGGgTtgAAggatGAttg
Deguchi *et al*., 1995 [35]	Mg1a, GGTTAACTTACC(T b )AGTGgCTTTgATCMg3 (inner), GTAATTAGTTACTCAGTAGA	Mg2, same as above
Totten *et al*., 2001 [36]	ModMgPa1, TGAAACcTTAACCCCTTGG	ModMgPa3, AGGGGTTTTCCATTTTTGC
Mena *et al*., 2002 [19]	MgPaW1, AAGTGGAGCGATCATTACTAAC	MgPaWR1, CCGTTGTTATCATACCTTCTGA
Jensen *et al*., 2004 [15]	MgPa-355F c, GAGAAaTACCTTgATGgTcaGCAA	MgPa-432R, GTTAATATCATATAAAGCTCT ACCGTTGTTATC

a ) Variable nucleotides are shown in lowercase.

b ) The nucleotide T is missing as a mistake in the report [35].

c ) Variation in positions underlined was also reported in reference [15].

## Discussion

The present study represents the first description of complete MgPa operon sequences for multiple *M. genitalium* clinical strains. Additionally, we provide sequences for all nine MgPars of an *M. genitalium* strain other than G37 and these data are used to assess MgPar sequence variability over time in clinical specimens. We have found that all MgPa operon sequences are different from each other among strains and also from the published G37 operon sequence as are all MgPars. Over the entire MgPa operon the four repeat regions exhibit extensive variation within and among strains while the non-repeat regions are relatively conserved, consistent with previous observations that the repeat regions are undergoing rapid genetic changes by recombination with MgPars, presumably due to immune or microenvironmental selection pressure [1], [10]–[13]. The first gene (MG190) of the MgPa operon is hypothesized to encode an enzyme controlling the homologous recombination of the MG191 and MG192 repeat regions with MgPars [10], [11]. The extremely high-level sequence conservation found for this gene in this study may reflect the necessity of sequence conservation in maintaining the function of this gene.

Previous studies of MgPa variation in vivo have focused on either the MG191 or MG192 repeat regions [10]–[13] and there has been no report on the sequence variability over the complete MgPa operon in vivo. In the present study we sequenced the complete MgPa operon in sequential specimens from each of two patients and identified intra-strain variation and segmental sequence shifts in one of the three MG191 repeat regions and in the MG192 repeat region between the first- and second-visit specimens in both patients (Figure 3). This observation shows that sequence change can occur in the MG191 and MG192 gene simultaneously within a time period as short as 10 days. All observed sequence shifts can be explained by homologous recombination with MgPars, consistent with previous studies by us and others [1], [10]–[13]. However, the MgPar sequences obtained were not adequate to determine if the recombination is due to gene conversion or gene cross-over.

In the present study we have presented a large set of MgPar sequences from clinical *M. genitalium* strains, including the complete set of nine MgPars obtained from the specimens of the patient 199. No MgPar sequence was identical between any two strains. This suggests that each strain may have a unique set of MgPars that have evolved independently in different strains. This finding is also consistent with the high-level variation in the MG191 and MG192 repeat regions among different strains. A striking finding in the data presented here is the nearly complete sequence homogeneity within strains in each the 43 MgPar regions studied. This observation contrasts sharply with the extensive MG191 and MG192 repeat region sequence heterogeneity within individual strains found in this study and described previously [10]–[12]. If gene cross-over had occurred, we would have expected the MgPar sequences to be highly heterogeneous within strains. These findings appear to support gene conversion as the most common recombination mechanism for the MgPa repeat region variation [11].

Our study of the genetic variation in the MgPa operon and MgPars not only provides new insights into the role of MgPa in the pathogenesis of *M. genitalium* infection as discussed above, but has important implications for the development of molecular diagnostic methods. Over the past two decades, most of the published diagnostic PCR assays for *M. genitalium* have relied on amplification of a region within the MgPa operon of the G37 type strain [1]. However, few studies have been able to take into account MgPa operon sequence variation in the selection of PCR primers as until now sequence data from multiple strains have not been available. In the present study we have demonstrated significant variation in some non-repeat regions of these genes in addition to the extensive variation in the four repeat regions (Figure 1). By comparing the sequences of the reported 19 primers with the complete MgPa operon sequences obtained in this study, we found that nine primers located in the non-repeat regions contained nucleotides in 1 to 5 variable positions. Two of the reported primers were located in the MG191 hypervariable repeat region B (primers MGS-2 and MgPa-903). These primers contain nucleotides corresponding to 11 and 19 variable sites, respectively (Table 3). The mismatches between the primers and the DNA template are likely to affect the efficiency of PCR amplification, thus giving rise to weak signals or false negative results, as has been demonstrated in studies involving the use of primers MGS-2 and MgPa-903 [1], [27], [28]. Caution should be exercised in interpreting the results of published studies in which these primers were used. It may be necessary to re-test the efficiency of PCR assays using these primers and cloned DNA templates containing known sequence variations. The availability of a large set of whole MgPa operon sequences as presented in this study should help develop new and better diagnostic PCR assays. The high-level sequence conservation in the MG190 gene as well as in the 3′-end of the MG192 gene suggests that these regions might be useful for PCR assay development.

Recognizing and understanding the genetic variation in the MgPa operon also has important implications for the development of molecular typing systems. The utility of the MgPa operon as a marker for *M. genitalium* genotyping has been well demonstrated in a typing system based on PCR and sequence analysis of the proximal region of approximately 280 bp from the MG191 gene [14]. This system offers high stability and excellent discriminatory power, and has been successfully used for confirming sexual transmission of *M. genitalium* [14], [16], [29] and for determining the identity of new clinical strains [10], [11], [16], [29]. The robust performance of this typing system is explained by the whole MgPa operon analysis presented here. As shown in Figure 1, the target for this typing system is located in a non-repeat region (so expected to confer stability within strains) but with significant variation among strains (necessary to give high discriminatory power). Another MgPa-based typing system [26] uses a target including almost the entire MG192 JKLM repeat region. Our current study as well as previous studies [11], [12] have found that this region undergoes rapid sequence shifts over time within strains, indicating that it is not suitable as the basis for an *M. genitalium* strain genotyping system. Based on the sequence variability of the whole MgPa operon in this study (Figure 1), we postulate that a few other non-repeat regions with high-level interstrain variation, particularly the two regions between the MG191 repeat regions B, EF and G (both of which contains both TTR variation and single-nucleotide polymorphisms), also could be useful for strain genotyping.

In summary, we have investigated the sequence variation in the whole MgPa operon and its chromosomal repetitive elements (MgPars) in clinical strains of *M. genitalium*. The presence of multiple hypervariable sites within the MgPa operon and the abundance of MgPars outside the MgPa operon as the donor sequences support the development of an efficient recombination system by this organism to generate numerous variants. Such variation may allow this organism to evade the host immune response and to adapt to diverse host microenvironments, thus establishing persistent infection. The availability of the large set of MgPa operon and MgPar sequence data generated from this study should facilitate further research on the pathogenesis of *M. genitalium* infection and the development of new diagnostic methods.

## Materials and Methods

### Ethics Statement

Written informed consent was obtained from human subjects and the study protocol was approved by the Institutional Review Board of the Louisiana State University Health Sciences Center.

### *M. genitalium* specimens

We used 13 axenic isolates of *M. genitalium* (Table 1). All isolates except for M2282 were cloned by standard filtration or limiting dilution cloning procedures [23], [25]. These isolates were grown in modified Friis medium containing horse serum [25]. In addition, we studied two sequential urine specimens obtained from each of two men from New Orleans with acute urethritis (patient no. 199 and 64). Genotypes of *M. genitalium* obtained from each patient were identical between the two sequential specimens [16], indicating that each patient was infected with a single *M. genitalium* strain. Genomic DNA was extracted using the Chelex 100 Resin (Bio-Rad) or the High Pure PCR Template Preparation Kit (Roche) as described elsewhere [15], [19].

### PCR and sequencing of the MgPa operon

The complete MgPa operon was amplified in up to six overlapping fragments using primers as shown in Table 4. PCR amplification was performed with the GeneAmp XL Long-range PCR kit (Applied Biosystems Inc) according to manufacturer's instructions. Initially, all PCR products were directly sequenced using internal primers. If there was any indication of mixed sequences present in the sequence chromatograms, the PCR products were cloned into TOPO vector (Invitrogen) and multiple plasmid clones were sequenced as we described elsewhere [11]. For specimens from each of the two New Orleans patients, all MG191 and MG192 repeat regions were sequenced after TOPO cloning. Complete MgPa operon sequences assembled from PCR fragments have been deposited in the GenBank under accession numbers FJ872584- FJ872592 and GU226196-GU226203. For regions of the operon genes showing intrastrain variations, only the most predominant sequence was used in the MgPa operon assembly.

**Table 4:** Primers used to amplify the MgPa operon and MgPar sequences.

Primer	Sequence (5′→ 3′)	Target (sequence location^a^)
189F1	AACAGAGCCAACACCA	MG189 (220412–220427)
906F	GGATAGAACTGAGGAGTAATG	MG190 (221409–221429)
976R	TATTATGTCCTCCCCCACCA	MG190 (221460–221479)
2113F	GAAAACCATACTGCCTTTGG	MG191 (222616–222635)
2235R	CTTGCGTTGTAATCCCAGATC	MG191 (222718–222738)
2833F	CAGATCTTCACTCCCTA	MG191 (223336–223352)
3087R	GGTTAGTTTGTTAGACCAGTTA	MG191 (223569–223590)
3942F	TTGTATAATGCCGCATTAC	MG191 (224445–224463)
4146R	CAACCACCAAGTTGAAACCC	MG191 (224630–224649)
5360F	CAACTCCTAAAACCCCACCA	MG192 (225876–225895)
5518R	AGCAGTAACATTCTTACTATCCTC	MG192 (220598–220621)
6959F	GGGGTGTTTGATGCGTTAG	MG192 (227462–227480)
227529R	GATCTGATCAGTTCTGGAAGGTAAACG	MG192 (227505–227531)
MG192A^b^	CACTAGCCAATACCTTCCTTGTCAAAGAGG	MG192 (225974–226003)
8730R	CACTCAGTAAGTGCCACTGC	3′-UTR^c^ (229214–229233)
1F8	GTTCAGTTGCTTTACTTGTCA	MgPar 1 (85501–85514)
4F1	CTGCAGTTAGTCAGTTTGCTG	MgPar 4 (213363–213383)
5F2	CTGAAGGGGAACAAAAAGCTG	MgPar 5 (228988–229007)
191R^d^	CCACCATTCACCTCCCCA	MgPars 1, 4 and 5 (86574–86591)

This list does not include primers for MgPars in our previous report [11].

a ) On the *M. genitalium* G37 genome sequence (GenBank accession number NC_000908).

b ) From the report of Musatovova *et al*. [26].

c ) 3′-untranslated region of the MG192 gene.

d ) Used with primers 1F8, 4F1 and 5F2 to amplify the 5′-portion of MgPars 1, 4 and 5, respectively.

### PCR and sequencing of MgPar regions

In an earlier study [11], we reported nine MgPar sequences from the two urine specimens of patient 199, which included complete sequences for MgPars 3, 6 and 8, and partial sequences for the remaining MgPars. In the present study, we extended all of these partial sequences to their full-length. We also determined the complete or partial sequence of all nine MgPars in the two sequential urine specimens of patient 64 and selected MgPars from five Danish isolates. PCR amplification of the MgPar regions was performed using previously published primers and conditions [11] except that four additional primers (Table 4) were used to amplify the 5′-portion of MgPars 1, 4 and 5 that had not been analyzed previously in these strains. PCR products were sequenced directly and/or after subcloning into TOPO vectors as described above. The MgPar sequences described in this study are available from GenBank under accession numbers EF117289-EF117301, FJ872560-FJ872583 and FJ872593.

### DNA sequence analyses

Sequence alignment and comparison were accomplished using the Clustal W algorithm (Slow-Accurate option) in Lasergene's MegAlign (DNASTAR) and the MacVector software (version 11.1.2, MacVector, Inc). Sequence similarity plot was generated by the SimPlot software version 3.5.1 [30] using default settings except that the sliding window size was changed to 100 bp.

### Evaluation of the sequences of the MgPa regions used in published diagnostic PCR and genotyping assays

At the time of writing this manuscript we found a total of 16 reported diagnostic PCR assays and 9 of these targeted different regions of the MgPa operon, as listed in Table 3. There were only a few genotyping systems reported for *M. genitalium*, and two of these systems were based on sequence variation found in different regions of the MgPa operon [14], [26]. To look for potential impact of MgPa sequence variation on these PCR and genotyping assays, we mapped all published primers and genotyping targets against a sequence alignment of the whole MgPa operon from the G37 type strain [4] and the 15 strains sequenced in this study.
